# Reinvigorating AMR resilience: leveraging CRISPR–Cas technology potentials to combat the 2024 WHO bacterial priority pathogens for enhanced global health security—a systematic review

**DOI:** 10.1186/s41182-025-00728-2

**Published:** 2025-04-02

**Authors:** Olalekan John Okesanya, Mohamed Mustaf Ahmed, Jerico Bautista Ogaya, Blessing Olawunmi Amisu, Bonaventure Michael Ukoaka, Olaniyi Abideen Adigun, Emery Manirambona, Olakulehin Adebusuyi, Zhinya Kawa Othman, Olanegan Gloria Oluwakemi, Oluwaseunayo Deborah Ayando, Maria Ivy Rochelle S. Tan, Nimat Bola Idris, Hassan Hakeem Kayode, Tolutope Adebimpe Oso, Musa Ahmed, M. B. N. Kouwenhoven, Adamu Muhammad Ibrahim, Don Eliseo Lucero-Prisno

**Affiliations:** 1 Department of Public Health and Maritime Transport, University of Thessaly, Volos, Greece; 2 Department of Medical Laboratory Science, Neuropsychiatric Hospital, Aro, Abeokuta, Ogun State Nigeria; 3 Department of Medical Laboratory Science, Chrisland University, Abeokuta, Nigeria; 4 Faculty of Medicine and Health Sciences, SIMAD University, Mogadishu, Somalia; 5 Department of Medical Technology, Institute of Health Sciences and Nursing, Far Eastern University, Manila, Philippines; 6 Center for University Research, University of Makati, Makati, Philippines; 7 Department of Medical Laboratory Services, State Hospital, Ede, Osun State, Nigeria; 8 Community and Clinical Research Division, First On-Call Initiative, Port Harcourt, Nigeria; 9 Department of Medical Laboratory Science, Nigerian Defence Academy, Kaduna, Nigeria; 10 College of Medicine and Health Sciences, University of Rwanda, Kigali, Rwanda; 11 Faculty of Pharmacy, University of Ibadan, Ibadan, Nigeria; 12 Department of Pharmacy, Kurdistan Technical Institute, Sulaimani, Kurdistan Region Iraq; 13 Department of Pharmacy, University of Ilorin, Ilorin, Nigeria; 14 Department of Public Health, Kwara State University, Malete, Nigeria; 15 Department of Nursing, University of the Philippines School of Health Sciences, Manila, Philippines; 16 Department of Public Health, Al-Hikmah University, Ilorin, Nigeria; 17 Department of Medical Laboratory Science, Oyo State Hospital Management Board, Oyo, Nigeria; 18 Department of Medical Laboratory Science, Federal Teaching Hospital, Ido-Ekiti, Nigeria; 19 Department of Physics, Xi’an Jiaotong-Liverpool University, Suzhou, China; 20 Department of Immunology, School of Medical Laboratory Science, Usmanu Danfodiyo University, Sokoto, Nigeria; 21 Department of Global Health and Development, London School of Hygiene and Tropical Medicine, London, UK; 22 Research and Development Office, Biliran Province State University, Naval, Leyte Philippines; 23 Research and Innovation Office, Southern Leyte State University, Sogod, Southern Leyte Philippines

**Keywords:** CRISPR–Cas technology, Antimicrobial resistance, WHO bacterial priority pathogens, Global health security, Diagnostic applications, Delivery mechanisms

## Abstract

**Background:**

Antimicrobial resistance (AMR) poses a global health threat, particularly in low- and middle-income countries (LMICs). Clustered regularly interspaced short palindromic repeats (CRISPR)–Cas system technology offers a promising tool to combat AMR by targeting and disabling resistance genes in WHO bacterial priority pathogens. Thus, we systematically reviewed the potential of CRISPR–Cas technology to address AMR.

**Methods:**

This systematic review adhered to the Preferred Reporting Items for Systematic Reviews and Meta-Analyses (PRISMA) guidelines. A comprehensive literature search was conducted using the Scopus and PubMed databases, focusing on publications from 2014 to June 2024. Keywords included “CRISPR/Cas,” “antimicrobial resistance,” and “pathogen.” The eligibility criteria required original studies involving CRISPR/Cas systems that targeted AMR. Data were extracted from eligible studies, qualitatively synthesized, and assessed for bias using the Joanna Briggs Institute (JBI)-standardized tool.

**Results:**

Data from 48 eligible studies revealed diverse CRISPR–Cas systems, including CRISPR–Cas9, CRISPR–Cas12a, and CRISPR–Cas3, targeting various AMR genes, such as *blaOXA-232, blaNDM, blaCTX-M, ermB, vanA, mecA*, *fosA3*, *blaKPC*, and *mcr-1,* which are responsible for carbapenem, cephalosporin, methicillin, macrolide, vancomycin, colistin, and fosfomycin resistance. Some studies have explored the role of CRISPR in virulence gene suppression, including enterotoxin genes, *tsst1*, and *iutA* in *Staphylococcus aureus* and *Klebsiella pneumoniae*. Delivery mechanisms include bacteriophages, nanoparticles, electro-transformation, and conjugative plasmids, which demonstrate high efficiency in vitro and in vivo. CRISPR-based diagnostic applications have demonstrated high sensitivity and specificity, with detection limits as low as 2.7 × 10^2^ CFU/mL, significantly outperforming conventional methods. Experimental studies have reported significant reductions in resistant bacterial populations and complete suppression of the targeted strains. Engineered phagemid particles and plasmid-curing systems have been shown to eliminate IncF plasmids, cured plasmids carrying *vanA*, *mcr-1*, and *blaNDM* with 94% efficiency, and restore antibiotic susceptibility. Gene re-sensitization strategies have been used to restore fosfomycin susceptibility in *E. coli* and eliminate blaKPC-2-mediated carbapenem resistance in MDR bacteria. Whole-genome sequencing and bioinformatics tools have provided deeper insights into CRISPR-mediated defense mechanisms. Optimization strategies have significantly enhanced gene-editing efficiencies, offering a promising approach for tackling AMR in high-priority WHO pathogens.

**Conclusions:**

CRISPR–Cas technology has the potential to address AMR across priority WHO pathogens. While promising, challenges in optimizing in vivo delivery, mitigating potential resistance, and navigating ethical-regulatory barriers must be addressed to facilitate clinical translation.

**Supplementary Information:**

The online version contains supplementary material available at 10.1186/s41182-025-00728-2.

## Introduction

The proliferation of antimicrobial resistance (AMR) threatens global public health security, jeopardizing decades of medical progress in the twenty-first century. This multifaceted phenomenon disproportionately burdens low- and middle-income countries (LMICs), causing high morbidity and mortality, with approximately 4.95 million deaths reported in 2019 due to AMR in bacterial pathogens [1]. The constant evolution of antimicrobial-resistant bacterial infections threatens to render modern medicine obsolete, bringing us back to a time when even small infections could be lethal. This trend in global health has necessitated strategic and innovative solutions targeting emerging drug resistance in infectious diseases [2]. The World Health Organization (WHO) released the Bacterial Priority Pathogens List (BPPL) in 2024 after periods of stringent surveillance and mapping worldwide since its inception in 2017. This release highlighted 15 families of globally alarming pathogenic bacteria, categorized into medium-, high-, and critical-risk groups [3]. In addition, the 2024 BPPL saw modifications from the 2017 reports, which included the incorporation of third-generation cephalosporin-resistant *Enterobacterales* as a freestanding unit in the critical priority category, underscoring their daunting threat. In addition, reclassifying carbapenem-resistant *Pseudomonas aeruginosa* (CRPA) infection from critical to high priority stresses the need to address the ravaging threat of AMR with utmost urgency [3].

**Fig. 1:**
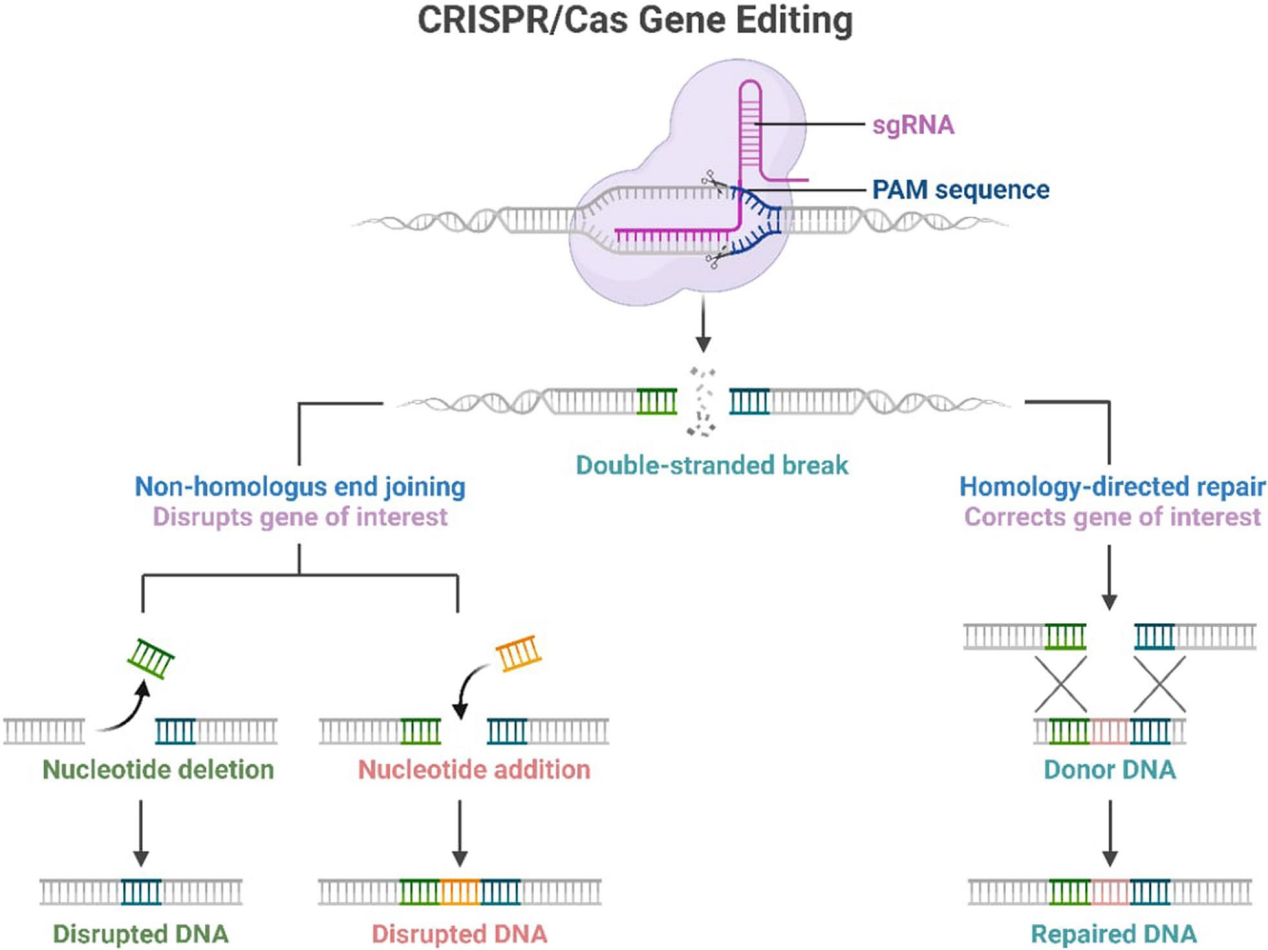
Molecular mechanism of CRISPR–Cas gene editing in bacterial cells [11]

Despite the growing body of literature on AMR, existing reviews have primarily focused on conventional therapeutic and antimicrobial stewardship strategies, with limited emphasis on clustered regularly interspaced short palindromic repeats (CRISPR)–Cas technology as an emerging gene-editing technology for alternative interventions [4, 5]. A comprehensive analysis linking this technology to priority bacterial pathogens and global health security remains unexplored [6, 7]. Existing knowledge highlights the escalating global health threat posed by AMR, especially in LMICs, where the burden is disproportionately high. Amidst the escalating peril of AMR, a breakthrough in the CRISPR–Cas system has rapidly transformed it into a versatile and target-specific technology that can be used as a tool for precise genetic editing [8]. Its potential to revolutionize the fight against AMR is profound, enhancing targeted interventions to fight antibiotic resistance in pathogens by selectively disabling the genes responsible for resistance, biofilm formation, pathogenicity, virulence, and bacterial viability (Fig. 1) [3, 8]. The specific gene recognition and targeted DNA cleavage characteristics of CRISPR/Cas technology can be utilized for pathogen detection and elimination of drug-resistant bacteria and genes, and hold promise as a new strategy for clinical diagnosis and treatment [9]. Compared to previous iterations, the 2024 BPPL is particularly relevant for CRISPR–Cas applications because of its refined categorization of priority pathogens, which facilitates targeted genetic interventions. This specificity enables the precise deployment of CRISPR-based antimicrobials and diagnostic tools, addressing the most urgent AMR threats [6, 10].

While CRISPR–Cas technology demonstrates the potential to combat AMR, its clinical usage is yet to be fully harnessed, as challenges abound, including the need to optimize delivery mechanisms, such as plasmid conjugation, bacteriophages, and nanoparticles, as well as the need to rectify possible mechanisms of interference in the configuration of this novel approach [3, 12]. Streamlining the delivery of CRISPR/Cas DNA cassettes (s) into the targeted bacterial population is pivotal, as is the development of multiplasmid conjugation systems for efficient CRISPR/Cas delivery, target DNA elimination, and plasmid replacement [13]. In addition to these regulatory hurdles, CRISPR/Cas technology raises ethical considerations in the context of genome modification, human and microbial subjects, and its potential application in infectious disease research and antimicrobial therapy [14, 15]. Issues such as unintended genetic alterations, horizontal gene transfer risks, and the ecological impacts of gene editing require thorough ethical scrutiny. In addition, debates surrounding the dual-use potential of CRISPR and its implications for biosecurity underscore the necessity of stringent regulatory frameworks to govern its application in AMR research and therapy [16, 17].

This review aims to assess the potential of CRISPR/Cas technology in combating AMR, with a focus on the WHO BPPL for 2024. This review highlights the critical role of CRISPR/Cas in reinvigorating AMR resilience. By focusing on the WHO BPPL for 2024, we explored the potential of this revolutionary tool to upscale infectious disease control measures and intensify the global health security. Redirecting this trajectory with tailored interventions prioritizing BPPL is a step towards achieving the 2030 Sustainable Development Goals (SDG). Through a comprehensive analysis of current research and emerging applications, we synthesized the transformative potential of CRISPR/Cas for combating AMR and 2024’s WHO Bacterial Priority Pathogens for Enhanced Global Health Security.

## Methodology

This systematic review aimed to assess the global safety and efficacy of the CRISPR/Cas system in addressing AMR. This review adhered to the Preferred Reporting Items for Systematic Reviews and Meta-Analyses (PRISMA) guidelines [18]. The PRISMA checklist for this review is provided in Supplementary File 1 [S1]. A comprehensive literature search was conducted in the Scopus and PubMed databases, ensuring alignment with the Medical Subject Headings (MeSH) terms for enhanced search accuracy. The keywords were refined using the MeSH database to include “CRISPR–Cas Systems,” “Antimicrobial Resistance,” “Bacterial Pathogens,” “Gene Editing,” and related terms. Boolean operators (“OR” and “AND”) were applied to optimize search sensitivity and specificity to retrieve all relevant studies. For Scopus, the following search query was used: TITLE-ABS-KEY (“CRISPR–Cas Systems” OR “CRISPR” OR “Gene Editing” AND “Antimicrobial Resistance” OR “AMR” OR “Bacterial Pathogens”) AND (LIMIT-TO (DOCTYPE, “ar”)) AND (LIMIT-TO (LANGUAGE, “English”)) AND (LIMIT-TO (SRCTYPE, “j”)). For PubMed, the search was conducted using the following query: (“CRISPR–Cas Systems” [MeSH Terms] OR “CRISPR”[Title/Abstract] OR “Gene Editing”[Title/Abstract]) AND (“Antimicrobial Resistance”[MeSH Terms] OR “AMR”[Title/Abstract] OR “Bacterial Pathogens”[Title/Abstract]) AND (English[Language]). The search strategy for each database included specific filters for peer-reviewed articles, English language, and human participants. A bibliometric search of the included publications was conducted to identify other important studies. The search was restricted to studies published between 2014 and June 2024, as CRISPR/Cas applications for AMR began gaining significant research attention in 2014, and to ensure the inclusion of the most recent and relevant data. Earlier studies primarily focused on the fundamental mechanisms of CRISPR immunity in bacteria rather than on therapeutic applications, making them less relevant to the objectives of this review.

### Eligibility criteria

Studies were included if they investigated the use of CRISPR/Cas technology for AMR, utilized a cross-sectional, cohort, or experimental study design, and were published in English between 2014 and 2024. We selected studies that reported quantifiable data or qualitative safety observations on the safety and efficacy of CRISPR/Cas. Studies that focused solely on theoretical modeling, in silico predictions without experimental validation, or applications unrelated to AMR were excluded, as were articles, such as case reports, reviews, editorials, letters, and commentaries.

### Study screening and selection

Two independent reviewers (OJO and BOA) screened the study titles and abstracts to confirm eligibility. A third reviewer (BMU) was consulted to resolve any discrepancies. Automation tools were not used during the screening process. After the elimination of duplicates, 48 studies met the inclusion criteria. Full-text screening was performed by the same independent reviewers, and discrepancies were discussed and resolved by a third reviewer. The studies included in this review were subjected to data extraction.

### Data extraction

The data were extracted independently by two reviewers from the 48 selected studies that met the eligibility criteria after full-text screening, as stated above. Information such as the author's name and year, study aims, CRISPR/Cas system used, target genes, delivery mechanism, dosage and administration protocols, efficiency and success rates were incorporated into the data extraction table.

### Outcomes and variables

The primary outcomes sought were the safety and efficacy of the CRISPR/Cas system in addressing AMR, measured by success rates in gene targeting and bacterial population reduction. The secondary outcomes included the efficiency of the delivery mechanisms and any reported adverse effects. Other variables collected included the study design, patient demographics, and funding sources.

### Risk of bias assessment

The risk of bias in the included studies was assessed using the Joanna Briggs Institute (JBI) standardized tool. Two independent reviewers assessed each study, and any disagreements were resolved through discussion with a third reviewer [19]. The JBI tool was applied based on study design-specific criteria, including a comprehensive assessment of the multiple domains. Each study was evaluated using the following criteria: clear inclusion criteria, detailed description of study subjects and setting, validity and reliability of exposure measurement, use of objective and standardized criteria for condition measurement, identification and management of confounding factors, validity and reliability of outcome measurement, and appropriateness of the statistical analysis. Based on these assessments, each study was assigned a final risk rating of low, moderate, or high, according to the JBI guidelines.

### Data synthesis and analysis

A thorough qualitative synthesis approach was used to evaluate and compile research results on the safety and effectiveness of the CRISPR/Cas system against resistant bacterial infection. The analysis considered the diversity of patient demographics, dosage schedules, and study designs. To achieve a full and transparent assessment, discrepancies in the literature were found, noted, and extensively addressed, particularly in terms of study outcomes. No meta-analysis was conducted because of the heterogeneity of the included studies**.**

## Results

### Overview of included studies

**Fig. 2:**
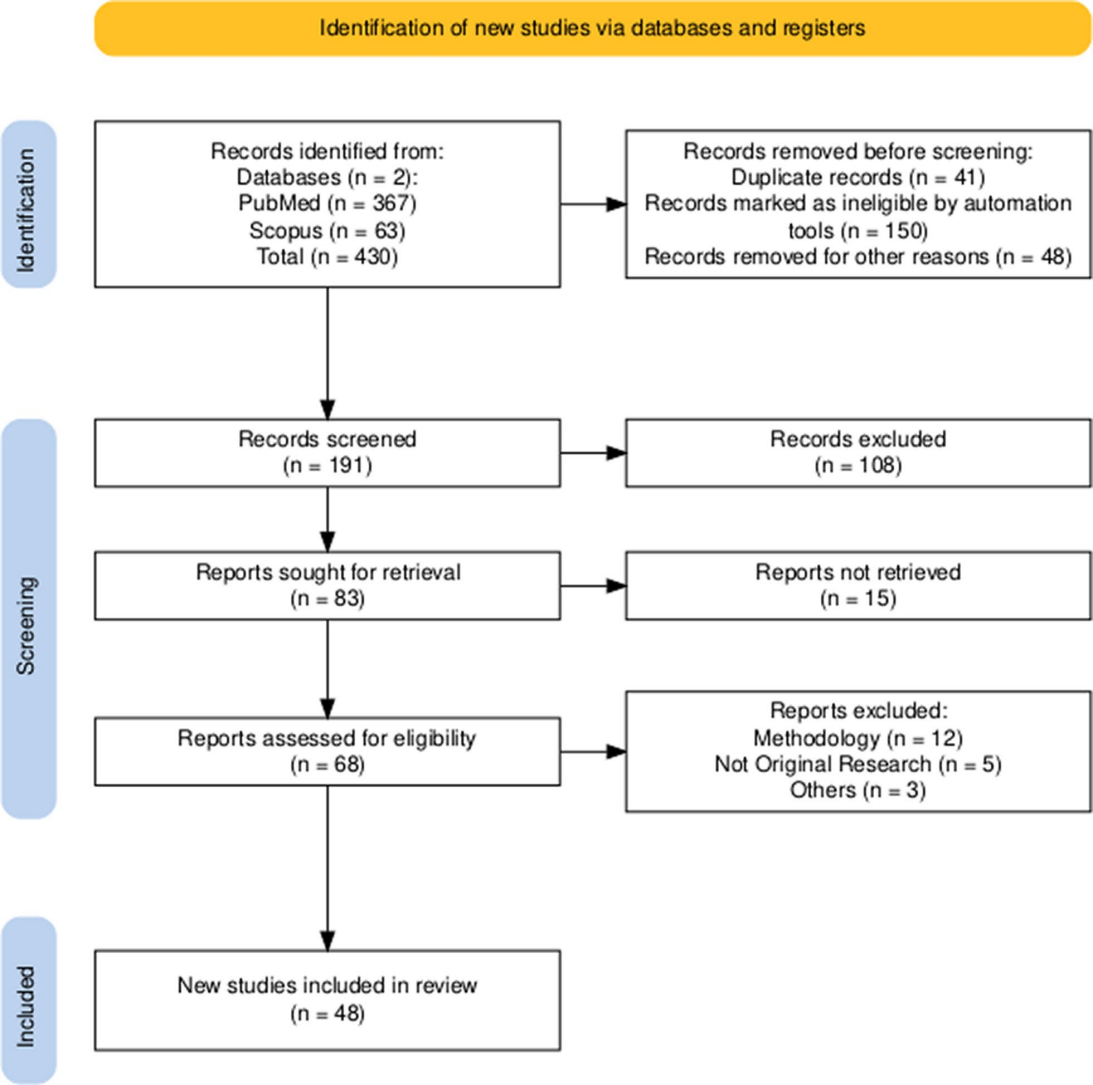
PRISMA flowchart of the included studies

This study evaluated 48 studies that investigated the use of CRISPR–Cas technology to address AMR using diverse study methods [20–68]. A PRISMA flowchart is presented in Fig. 2, illustrating the search and selection processes. Initially, 430 records were identified in the literature search. After removing duplicates and screening titles and abstracts, 68 studies were assessed for full-text eligibility. Of these, 48 studies met the inclusion criteria and were included in this review. The 48 included studies encompassed a variety of experimental designs, including 28 experimental studies, 3 comparative studies, 2 lab-based experiments (in silico analysis and proof-of-concept research), 3 observational studies, 2 investigative studies, 2 in vivo investigations, and 6 genome sequencing analyses. A summary of the salient information regarding the use of the CRISPR/Cas system to combat bacterial AMR is presented in Table 1.

**Table 1:** CRISPR/Cas system applications in bacterial and AMR studies

Author’s name and year	Study design	Aim	CRISPR/Cas system used	Target genes	Delivery mechanism	Dosage and administration protocols	Efficiency and success rate
[20]	Experimental assay	To propose a PCR-coupled CRISPR/Cas12a-based fluorescence assay for detecting NDM-producing genes (blaNDM) in bacteria	CRISPR/Cas12a	New Delhi metallo-β-lactamase producing genes (bla_NDM_)	PCR-coupled CRISPR/Cas12a-based fluorescence assay	100 nM Cas12a, 100 nM gRNA, 100 nM ssDNA-FQ reporter, 12 µL PCR product, 9 µL NEBuffer 2.1, total 90 µL with nuclease-free water, fluorescence measured for 30 min at 37 °C	The detection limit of 2.7 × 100 CFU/mL, 100 times better than conventional PCR with gel electrophoresis
[21]	Experimental amplification-free electrochemical CRISPR/Cas biosensor study	To develop an amplification-free electrochemical CRISPR/Cas biosensor for the detection of methicillin-resistant Staphylococcus aureus (MRSA)	CRISPR/Cas12a	mecA gene of MRSA	Electrochemical biosensor utilizing silver metallization	50 nM Cas12a enzyme and gRNA, 1 µL target gene, washing, immersed in 100 mM AgNO3 in 20 mM HEPES and 100 mM NaNO3 [pH 7.4) for 30 min	Detection and quantitation limits of 3.5 fM and 10 fM, respectively, linearity over five orders of magnitude (from 10 fM to 0.1 nM)
[22]	Experimental study	To reverse antibiotic resistance in E. coli by programming CRISPR–Cas9 with spacers targeting blaCTX-M genes and their promoters	CRISPR–Cas9	(blaCTX-M-15 and -55) and (blaCTX-M-14, − 27, − 65, and − 90)	non-replicative phagemid particles	Varied multiplicities of infection (MOI), ranging from 0.1 to 100	The CRISPR–Cas9 system successfully re-sensitized E. coli to third-generation cephalosporins by targeting blaCTX-M genes, reducing the ratio of resistant cells by 3 to 4 log10, but spacers targeting promoters did not yield significant re-sensitization
[23]	Comparative analysis of CRISPR–Cas systems across ESKAPE + C pathogens	The study aims to annotate and compare the CRISPR–Cas systems in the ESKAPE pathogens (*Enterococcus faecium, Staphylococcus aureus, Klebsiella pneumoniae, Acinetobacter baumannii, Pseudomonas aeruginosa,* and *Enterobacter species)* and *Clostridium difficile*, and investigate the relationship between the presence of CRISPR–Cas systems and antimicrobial resistance	Various CRISPR–Cas systems (e.g., Type I-B in C. difficile)	Not specified	Not specified	Not specified	The study found that CRISPR–Cas-containing isolates tend to have more antimicrobial resistance genes for four of the pathogens (*A. baumannii, E. faecium, P. aeruginosa,* and *S. aureus*), suggesting a potential link between CRISPR–Cas systems and the acquisition of antimicrobial resistance
[24]	Experimental design	The study aims to employ a CRISPR–Cas9-mediated pCasCure plasmid-curing system to precisely remove specific IncF plasmids from multidrug-resistant (MDR) extraintestinal pathogenic Escherichia coli (ExPEC) clones, and investigate the broader roles of these IncF plasmids in the success of MDR ExPEC clones beyond antimicrobial resistance	CRISPR–Cas9	IncF plasmids ranging from 51 to 167 kb in size	pCasCure plasmid-curing system	Not specified	Successful plasmid curing was confirmed through PCR and whole-genome sequencing, which showed the absence of the target IncF plasmids without any additional off-target mutations. Curing of the IncF plasmids restored susceptibility to various antibiotics
[25]	Genome sequencing and analysis of CRKP isolates	To reveal and assess the genomic characteristics of blaNDM-carrying CRKP clinical isolates from a university hospital in Thailand	Identified CRISPR–Cas region in the CRKP9 isolate with 28 distinct spacer sequences	blaNDM-1, blaOXA-232, blaCTX-M-15	Not specified	Not specified	Not specified
[26]	Experimental Validation Study	To adapt a CRISPR–Cas9 system named pRE-FOSA3 to restore the sensitivity of a fosA3 + Escherichia coli strain	CRISPR–Cas9	fosA3	pRE-FOSA3 plasmid	Not specified	gRNA_195 exhibited 100% efficiency in resensitizing the bacteria to fosfomycin
[27]	Experimental validation study	To design a mobile, broad host-range CRISPR–Cas9 expression system that can block AMR gene uptake in multiple species	CRISPR–Cas9	aacC1 (gentamicin resistance gene)	pKJK5::csg, a broad host-range IncP1 plasmid	Not specified	pKJK5::csg[aacC1] reduced transformation efficiency of the targeted plasmid pHERD30T by at least four orders of magnitude compared to the non-targeting control in E. coli. pKJK5::csg[aacC1] reduced the proportion of E. coli K12 recipients carrying the resident pHERD30T plasmid from 58.8% to 25.6% after conjugation. < br >—In a range of bacterial isolates, pKJK5::csg[aacC1] reduced transformation efficiency of the targeted plasmid by at least two to three orders of magnitude compared to the non-targeting control
[28]	Whole genome sequencing and genetic analysis	To map genes associated with antimicrobial resistance (AMR) and virulence factors, and to identify multilocus sequence types (MLST) of carbapenem-resistant Acinetobacter baumannii (CRAb)	Not mentioned	Various antimicrobial resistance (AMR) genes including bla OXA-72, ade genes (RND: adeFJK, adeLN & adeR), and SMR (abeS)	Whole-genome sequencing (WGS) using Illumina MiSeq	Not applicable	The study successfully identified 23 antibiotic-resistance genes in all strains of A. baumannii, with 12 shared by all three strains
[29]	WGS-based surveillance study	To perform whole genome sequencing (WGS) analysis of a diverse set of antimicrobial-resistant Staphylococcus aureus isolates from ready-to-eat (RTE) food in various geographic regions of Russia, characterizing their clonal structure, resistance and virulence determinants, plasmid replicon sequences, and CRISPR/Cas systems	CRISPR/Cas type IE and I systems	Various antimicrobial resistance and virulence genes, including enterotoxin genes, tsst1 gene, beta-lactam resistance genes (blaZ), methicillin resistance gene (mecA), and vancomycin resistance gene (vanB)	Whole genome sequencing (WGS)	Not applicable	The study identified diverse genetic lineages, resistance determinants, and virulence genes in the isolates, indicating a significant public health threat. Approximately 40% of the isolates carried at least one enterotoxin gene, and 70% of MRSA isolates carried the tsst1 gene
[30]	Experimental study	To investigate the role of mobile genetic elements (MGEs) in the spread of antimicrobial resistance (AMR) and the conflict between MGEs mediated by CRISPR systems among ESKAPE pathogens	Various types including I-C, I-E, III-A, IV-A1, IV-A3	Various antimicrobial resistance genes and virulence genes	MGEs including plasmids, ICEs/IMEs, and prophages	Not specified	Not quantified in terms of detection limits or success rate but highlights the pervasive association of AMR genes and anti-CRISPRs with the ESKAPE mobilome, showing effective gene flow across MGEs
[31]	Genome-wide analysis	To investigate the influence of the DNA phosphorothioation (PT) restriction-modification (R-M) system on the antimicrobial resistance (AMR) of pathogenic bacteria	CRISPRCasFinder v4.2.20	AMR genes	Prediction of CRISPR–Cas systems and R-M systems in bacterial genomes using bioinformatics tools (Restriction-ModificationFinder and CRISPRCasFinder)	Not specified	Identification of the correlation between the presence of PT R-M clusters and the abundance of AMR genes in different bacterial strains
[32]	WGS-study	To perform whole genome sequencing to identify drug resistance genes in Streptococcus anginosus strain 47S1	Not specified	Multiple resistance genes	Not specified	Not specified	High resolution of resistance profiles through shotgun sequencing
[33]	Hybrid genome sequencing and analysis	To explore the pangenome dynamics and phylogroup-specific characteristics of Pseudomonas aeruginosa, focusing on genome size, accessory gene content, AMR, and defence systems	CRISPR–Cas systems were observed in the context of their influence on genome size and the prevalence of AMR and defence systems	Not explicitly mentioned	Not applicable	Not applicable	Provides insights into the association between CRISPR–Cas systems and genome size variation, as well as their impact on AMR and defence system prevalence
[34]	Cross-sectional study	To characterize L. monocytogenes isolates from the beef production chain regarding their STs, virulence factors, AMR genes, etc	CRISPR–Cas system (Class1-Subtype-I-B_1)	fosX, vga(G)	Not specified	Not specified	Not specified
[35]	CRISPR–Cas9 re-sensitization model study	Re-sensitization of E. coli to antibiotics by targeting blaCTX-M AMR genes	CRISPR–Cas9	blaCTX-M group 1 (blaCTX-M-15, − 55) and group 9 (blaCTX-M-14, − 27, − 65, − 90)	Phagemid particles (ΦRC319)	Dose-dependent from 0.1 to 100 MOI	3 log10 to 4 log10 reduction of the ratio of resistant cells for spacers targeting internal sequences
[36]	Cross-sectional observational study	Evaluate the synergistic effects of fosfomycin in combination with other antimicrobial agents against blaNDM-harboring carbapenem-resistant Escherichia coli (CREC) and to characterize the whole-genome and plasmid sequences of these pathogens	Not applicable	blaNDM-1	Not applicable	Fosfomycin combined with aminoglycosides, colistin, tigecycline, sitafloxacin, and ciprofloxacin using the checkerboard method	Synergistic effects were observed in combinations against blaNDM-1-harboring CREC isolates. The study demonstrated that the whole-genome and plasmid sequences might help control the spread of these pathogens
[37]	Cross-sectional microbiiological study	To genetically characterize MDR-HvKp ST2096 isolates harboring hybrid plasmids carrying both antimicrobial resistance (AMR) and virulence genes	Type IV-A3 CRISPR–Cas system	Virulence genes: rmpA2, iutA, iucABCD AMR genes: blaNDM-5, aadA2, armA, blaOXA-1, msrE, mphE, sul1, dfrA14	The study does not specifically mention the delivery mechanism for the CRISPR–Cas system, but it mentions the presence of hybrid plasmids in K. pneumoniae isolates	Not applicable	The study demonstrates the presence of hybrid plasmids encoding both virulence and resistance traits but does not specify efficiency or success rates in quantitative terms
[38]	In vivo investigation	To exploit a native CRISPR–Cas3 system for curing high-risk IncFII plasmids in MDR K. pneumoniae	CRISPR–Cas3	IncFII plasmids	Conjugation	In vitro and In vivo plasmid curing assays, including Galleria mellonella infection model	High plasmid curing efficiency In vitro (8-log decrease) and In vivo (~ 100% curing) in a Galleria mellonella infection model
[39]	Comparative genomic analysis of CRISPR systems	To analyze the CRISPR–Cas system of S. Typhi isolates from South Asian countries, focusing on its diversity and potential use as biomarkers, particularly related to antimicrobial resistance (AMR)	S. Typhi CRISPR–Cas system	Spacers targeting bacteriophages and plasmids, including Ts32g and Ts32i	Not applicable	Not applicable	The study identified strong correlations between variations in the S. Typhi CRISPR–Cas system and AMR status, with specific markers linked to XDR isolates
[40]	Experimental study	To explore the function and mechanism of CRISPR/Cas systems in *E. faecalis* T11, focusing on mutant generation and analysis	CRISPR3–Cas and CRISPR2 systems in *Enterococcus faecalis* T11	CRISPR3 cas9 and CRISPR3 spacer 6	Electroporation transformed plasmid	Not Specified	Sequencing confirmed success in generating desired deletions and mutations, demonstrating effective implementation of the CRISPR/Cas system through the generation of mutants and complementation strains
[41]	Comparative defense mechanism analysis	To identify and characterize a bacterial CreTA in Acinetobacter's subtype I-F CRISPR–Cas system	AYE CRISPR–Cas	*aac3* and *oxa23*	Conjugative plasmids and electro-transformation methods	300 ng plasmids for electro-transformation, IPTG induction (0.5 mM) for CRISPR expression	The transformation efficiency was determined using CFU/μg plasmid DNA, with variable success rates indicated by colony counts and PCR validations
[42]	Experimental Study	Re-sensitize bacteria to carbapenems and reduce blaKPC-2 gene transmission	Prokaryotic CRISPR–Cas9	blaKPC-2	Plasmid pCas9-sgRNA(blaKPC-2) transformation into E. coli	10 µL of pCas9-sgRNA plasmid	Efficient clearance of blaKPC-2-harboring plasmids, restored antibiotic susceptibility after plasmid clearance by the CRISPR–Cas9 system
[43]	Experimental study	To develop and optimize a broad-host-range plasmid for CRISPR–Cas-mediated gene-curing to combat antimicrobial resistance	CRISPR–Cas12f	mcr-1 and blaKPC	pQ-mini plasmid derived	Not explicitly stated	pQ-mini shows high transfer efficiency and remarkable curing efficiencies for mcr-1 and blaKPC genes, comparable to the pCasCure system
[44]	Investigative study	Understand how Shigella strains regulate CRISPR–Cas activity and the impact of insertion sequences (IS) on antimicrobial resistance gene acquisition	CRISPR Cas in Shigella	cse2, cas6e, and cse1–cas3	Electrotransformation of a resistance plasmid	Not specified	IS600 insertion significantly reduced the relative expression of the cse2 gene, indicating an impact on CRISPR–Cas activity
[45]	Laboratory-based study	To develop and assess the effectiveness of the pIS26-CRISPR/Cas9 system for plasmid curing and evaluate its impact on antimicrobial resistance	CRISPR/Cas9 system integrated into the pIS26 plasmid	mcr-1, blaKPC-2, blaNDM-5, and replication genes of various plasmids (IncX4, IncI2, IncHI2)	Conjugative plasmid transfer through bi-parental mating	Not stated	Curing efficiency of targeted genes and plasmids was 100% ± 0 in multiple strains
[46]	Experimental laboratory study	To investigate the genetic mechanisms and responses of *F. novicida* to polymyxin B treatment	Cas9-dependent CRISPR–Cas system	Cas9 regulatory axis deletion mutants, FTN_1254, FTN_0109 mutants	Gene deletion mutants were constructed by allelic exchange	Not stated	The study found that the Cas9-dependent CRISPR–Cas system improves envelope integrity and resistance to membrane stressors, including antibiotics, crucial for evading the host's innate immune AIM2/ASC inflammasome during infection
[47]	Experimental study	To develop a tigecycline- and colistin-resistant bacteria resensitization system using efficient DNA damage from CRISPR-Associated Protein 9 nucleases	pCas/Ind and pCas/Con plasmids	glnA (glutamine synthetase), blaNDM-1, mcr-1, tet(X4)	Plasmid construction, transformation, and conjugation assays	In vitro: 100 µL competent cells with 100 ng pCas/Con and 50 ng psgRNA In vivo: 5 × 10^8 or 5 × 10^9 CFU donor strain, 20 mg/kg tigecycline or colistin	The study showcases the effectiveness and adaptability of CRISPR–Cas systems as potent antimicrobials in resensitizing tet(X4)- and mcr-1-mediated, tigecycline- and colistin-resistant strains, paving the way for the development of CRISPR-based tools for selective bacterial pathogen elimination
[48]	Experimental study	To study the relationship between CRISPR/Cas systems and multidrug resistance in *Escherichia coli*	CRISPR3 and CRISPR4	csy1 and cas1 for Type I-F CRISPR system rpsL for RT-PCR control	RT-PCR kit for csy1 and cas1 expression	Not specified	The CRISPR I-F system, associated with the B2 phylogroup, is overrepresented in the susceptible group compared to the MDR group. RT-PCR showed Type I-F cas genes were expressed, suggesting the CRISPR I-F system is more prevalent in antimicrobial-susceptible E. coli
[49]	Experimental study	To develop a user-friendly, cost-effective biosensor for swift detection of the macrolide resistance gene ermB in wastewater, thereby facilitating community-wide assessment	CRISPR/Cas12a	ermB Gene	Enzyme trans-cleavage activity coupled with visual assays	DNA polymerase to amplify the ermB gene	The ermB gene can be detected with a limit of detection as low as 2.75 × 10^3 copies/μL using fluorescence and lateral flow assays, demonstrating excellent selectivity and a detection time of 2 h
[50]	Experimental study	to determine whether the CRISPR/Cas9 system in *M. gallisepticum* was functional and whether it could be utilised to introduce mutations into the *M. gallisepticum* genome using synthetic CRISPR arrays carried on an *oriC* plasmid	Endogenous CRISPR/Cas System Arrays; Three constructs (pK1-CRISPR, pK-CRISPR-1, pK-CRISPR-2)	ksgA Gene	Plasmid construction	Not specified	Enhances the comprehension of the M. gallisepticum CRISPR/Cas system, potentially aiding in the creation of genetically modified tools
[51]	Experimental study	To explore the evolutionary history of CRISPR–Cas subtype I-Fb in *A. baumannii* and determine its genetic relatedness among a group of CRISPR-positive clinical isolates using comparative sequence analysis of spacer arrays	CRISPR–Cas subtype I-Fb system	cas1 gene (920 bp) csy1 gene (1251 bp) csy4 gene (615 bp) Concatenated MLST sequences (2976 bp)	Not specified	Not specified	The efficiency and success rate of detecting the CRISPR–Cas subtype I-Fb system were reflected in the results, showing that all 74 isolates included in the study carried the system
[52]	In-silico analysis	To investigate the presence, distribution, and characteristics of the CRISPR–Cas system in *A. baumannii* genomes and explore its potential association with resistance genes and its role in protecting against phage infections	CRISPR–Cas I-F1 subtype	CRISPR–Cas system itself	bioinformatics tools and database queries	Not applicable	Success is indicated by the identification of variations in CRISPR–Cas system architecture and its potential association with resistance genes
[53]	Experimental study	To create a suicide plasmid using CRISPR/Cas9 to target and eliminate the mcr-1 gene in various bacterial strains, evaluating its efficacy in curing plasmids	pISApl1-CRISPR/Cas9	mcr-1	Conjugation	Not specified	The transposon-associated CRISPR/Cas9 system could be a potential therapeutic tool for controlling the spread of mcr-1 resistance in clinical pathogens
[54]	Experimental study	To engineer pRK24 and pBP136kan by introducing Cas9 and gRNAs specific for conserved sequences in EPEC and EHEC genes, S. enterica ssaN genes, and plasmid-borne antibiotic resistance-blaCMY-2 gene	CRISPR/Cas9	eae ssaN blaCMY-2	Plasmid delivery	Not applicable	The study suggests that conjugative CRISPR–Cas9 antimicrobials can protect against enteric pathogens and reduce antibiotic resistance without disrupting the normal microbiota
[55]	Experimental study	To reduce the spread of the v*anA* gene by curing the *vanA*-harboring plasmid of vancomycin-resistant using the CRISPR–Cas9 system	CRISPR–Cas9 system	vanA gene	Transformation assay	Plasmid Transformation Conditions	The study demonstrated that CRISPR–Cas9 effectively achieved plasmid clearance and reduced antimicrobial resistance by blocking the horizontal transfer of plasmid carrying vanA
[56]	Experimental approach	To investigate how a target *E. coli* strain can escape killing by episomally-encoded CRISPR–Cas9 antimicrobials	CRISPR–Cas9 system with plasmids containing SpCas9 and gRNA	vanA resistance gene of E. coli	Plasmid transformation and construction	1 ng/µL plasmid DNA at 1.8 kV, followed by recovery in SOC media for 1 h	Most spontaneous mutations that rapidly confer resistance to CRISPR-induced killing are insertions and deletions in the cas9 gene and its regulatory elements, and multiple target sites of the gRNA prevent their selection
[57]	Experrimental study	To find out the relationship between cas9 gene and antimicrobial resistance in Campylobacter jejuni NCTC11168	CRISPR/Cas9 system	Cj1523c (Cas9)	Electrotransformation	Not specified	CRISPR–Cas system plays a role in the enhancement of antimicrobial resistance in C. jejuni
[58]	Experimental study	To establish efficient genetic tools and a CRISPR–Cas9 system for precise genome editing and functional genomics in Eubacterium limosum	CRISPR–Cas 9	Genes within the Wood-Ljungdahl pathway and the fructose-PTS system in E. limosum	Inducible promoter	Not specified	CRISPR–Cas9 system achieved 100% efficiency in manipulating target genes. CRISPR interference effectively reduced the expression of several genes in the Wood-Ljungdahl pathway and fructose-PTS system
[59]	Experimental study	To investigate how the interplay between CRISPR–Cas genome defence and antibiotic selection for mobile genetic elements shapes In vitro* E. faecalis* populations	CRISPR2	ermB	Conjugation and electroporation	Not stated	Forced maintenance of CRISPR targets can lead to a fitness cost that can be utilized to modify diverse *E. faecalis* populations
[60]	Experimental study	To develop a simple, rapid, sensitive, and specific detection platform for P. aeruginosa infection diagnosis	CRISPR–Cas12a (Cpf1)	*oprL* gene	combination of CRISPR–Cas12a and specific CRISPR RNAs (crRNAs))	Not specified	The P. aeruginosa–CRISPR–RPA assay demonstrated reliability for P. aeruginosa detection, as clinical samples showed consistent results with the initial microfluidic chip method, proving its effectiveness
[61]	Proof-of-concept study	To demonstrate that CRISPR–Cas9-mediated plasmid-curing and resistance gene elimination can effectively resensitize CRE to carbapenems	CRISPR–Cas9 system	blaKPC, blaNDM, blaOXA-48	Electrotransferred pCasCure plasmid	Not specified	pCasCure achieved 94% curing efficiency for targeted carbapenemase genes and plasmids, reducing MIC values by over eightfold in all tested isolates, except for IS1R-mediated recombination escape
[62]	Experimental study	To showcase the use of a nonviral, polymer-derivatized CRISPR–Cas9 system for genome editing, enhancing antimicrobial efficacy by reducing selective pressure	CRISPR–Cas9 system	mecA gene	Nanosized CRISPR complexes (Cr-Nanocomplex)	The Cr-Nanocomplex	The Cr-Nanocomplex demonstrated higher efficiency in genome editing compared to native Cas9 complexes or conventional lipid-based systems, with successful delivery and editing of the mecA gene in MRSA
[63]	Experimental study	To determine if the CRISPR–Cas csy1 gene affects antibiotic resistance in *Acinetobacter baumannii* and to explore the role of CRISPR–Cas in bacterial drug resistance	I-Fb CRISPR–Cas	csy1 gene	RecAb homologous recombination system	Not specified	The AB43Δcsy1 mutant strain showed increased antibiotic resistance, indicating the csy1 gene's role in inhibiting antimicrobial resistance, and the complete CRISPR–Cas system effectively prevented bacterial resistance development In vitro
[64]	Experimental study	To develop and demonstrate the use of CRISPR–Cas9 for targeted gene disruption in *R. toruloides*	CRISPR–Cas9 system	URA3	Transformation	Not specified	The initial editing efficiencies were low, but optimization increased them 364-fold to 0.6%, reaching 50% CAR2 deletion efficiencies, and multiplexed gene editing was successfully demonstrated by disrupting both CAR2 and URA3
[65]	Experimental study	To use CRISPR/Cas9 to target and disrupt specific sequences in ESBL-producing *E. coli* to restore antibiotic sensitivity	CRISPR/Cas9	TEM- and SHV-type ESBLs	Not specified	Not specified	CRISPR/Cas9 system was shown to re-sensitize MDR cells to antibiotics, even when resistance is mediated by genes on the same plasmid as the target genes
[66]	Experimental study	To create a CRISPR–Cas-based antimicrobial system for reducing antibiotic resistance in *E. faecalis* populations and assess its efficacy in a murine model	Type II CRISPR–Cas system	Antibiotic resistance determinants in *E. faecalis*	pheromone-responsive conjugative plasmid	Not stated	CRISPR–Cas antimicrobial plasmids significantly reduced antibiotic-resistant *E. faecalis *In vitro and In vivo, demonstrating a significant decrease in resistant strains. In addition, *E. faecalis* donor strains with the CRISPR–Cas system were immune to resistance determinants uptake
[67]	Experimental study	To create a host-independent conjugative plasmid using a CRISPR/Cas9 system to remove mcr-1 plasmids from bacteria	CRISPR/Cas9 system	Plasmids carrying the mcr-1 gene	A host-independent conjugative plasmid	Not specified	The CRISPR/Cas9 system successfully removed plasmids containing mcr-1 from bacteria, restoring polymyxin sensitivity. The recipient bacteria also gained immunity against mcr-1, proving its effectiveness in combating colistin resistance
[68]	Experimental study	To utilize CRISPR–Cas systems for the targeted removal of specific bacterial strains or species from mixed cultures	Type I-E CRISPR–Cas system	Specific bacterial strains	Not specified	Not specified	CRISPR–Cas systems can effectively target and remove individual bacterial strains or species from mixed cultures, demonstrating high selectivity and programmability, a feat not achievable with traditional antibiotics or bacteriophages

### CRISPR/Cas system and target genes

Diverse CRISPR/Cas systems have been used in the studies reviewed. The systems used include the CRISPR–Cas12a system used in five studies [20, 21, 43, 49, 60]**.** The CRISPR–Cas9 system (19 studies) [22, 24, 26, 27, 35, 42, 45, 46, 53–58, 61, 62, 64, 65, 67]**,** CRISPR–Cas3 system (2 studies) [37, 39]**,** various CRISPR–Cas systems (15 studies) [23, 25, 29, 31, 33, 34, 37, 39, 41, 44, 50, 51, 63, 66, 68]**,** CRISPR–Cas12f system (1 study) [42], and CRISPR–Cas 1 (1 study) [50]. These systems are specific for functions, such as gene editing, versatility of SpCas9 and gRNA plasmids, efficacy in bacterial immunity, and genome regulation. Some studies have examined CRISPR–Cas systems in specific bacterial strains, such as *Enterococcus faecalis* T11 and *S. typhi*, and CRISPR2 systems, providing insights into their applications in various microbial contexts. A review of the target genes revealed a broad spectrum of genes linked to resistance mechanisms and virulence. Focusing on genes associated with antibiotic resistance, the New Delhi metallo-β-lactamase producing gene (*blaNDM*) was reported in most studies reviewed [20, 25, 36, 37, 45, 47, 61]. Notably, it induces resistance to carbapenems. The *mecA* gene, associated with methicillin resistance in *Staphylococcus aureus*, and vancomycin-resistant genes have also been reported [21, 29, 62]. Other target genes include the *blaOXA-232* gene associated with resistance against oxyimino-cephalosporins [25], resistance genes from the *blaCTX-M* group [36], *fosA3* gene associated with fosfomycin resistance [26], *aacC1* gene responsible for gentamicin resistance [27], and *ade* and *SMR* genes [28]. Virulence factors, such as enterotoxin genes, *tsst1, rmpA2, iutA*, and *iucABCD*, have also been identified [29, 37]. The CRISPR–Cas system components were specifically outlined, and genes such as *cse2, cas6e, cas1*, and *cas3* were examined for their roles in function and regulation [44].

### Delivery mechanisms and administration protocols

Various techniques have been developed for CRISPR–Cas delivery and analysis. Common methods include PCR-coupled fluorescence assays and electrochemical biosensors [20, 21]. Engineered lambda phages and plasmids, such as pCasCure and pRE-FOSA3, are often used to introduce CRISPR components into bacterial systems [22, 24, 26, 30, 35, 37, 39, 41, 43, 47, 67]. Whole-genome sequencing (WGS) using Illumina MiSeq and other sequencing approaches provides comprehensive genomic data for analysis [24, 28]. Bioinformatics tools, such as Restriction-ModificationFinder and CRISPRCasFinder, are used to predict and analyze CRISPR–Cas and restriction-modification systems in bacterial genomes [31]. Conjugation and electroporation are common delivery methods that facilitate the transfer of CRISPR systems into target cells. Plasmid transformation, construction, and transformation assays, as well as nanosized CRISPR complexes, have also been used for this purpose. Techniques such as phagemid particles, conjugative plasmids, and bioinformatics-based approaches have also been used to explore CRISPR–Cas functionalities at various dosages and administration protocols, focusing on diverse experimental needs. For CRISPR–Cas12a assays, 100 nM concentrations of Cas12a, gRNA, and ssDNA-FQ reporter were used along with the PCR product and NEBuffer 2.1. For biosensor applications, 50 nM concentrations of Cas12a enzyme and gRNA were used, followed by silver metallization. For phage application, a multiplicity of infection (MOI) of 10 was used to suppress enterohemorrhagic *E. coli* (EHEC) growth for up to 18 h. For plasmid transformation, 300 ng of plasmid DNA was typically used, with induction using 0.5 mM IPTG for CRISPR expression. Other methods include varying MOIs, plasmid curing assays, and in vivo infection models.

### Efficiency and success outcomes

Several studies have demonstrated enhanced sensitivity of CRISPR-based detection methods, such as a PCR-coupled fluorescence assay with a detection limit of 2.7 × 10^2 CFU/mL [21]. These systems demonstrated detection and quantitation limits of approximately 10 fM, with linearity spanning five orders of magnitude. In antimicrobial applications, CRISPR–Cas systems have shown nearly 100% bactericidal potential against EHEC without affecting other *E. coli* strains and no resistance to El phages. The use of CRISPR–Cas systems in plasmid curing has also shown notable success, with high plasmid curing efficiency demonstrated in vitro (8-log decrease) and in vivo (~ 100% curing) using a *Galleria mellonella* infection model [24, 38].

CRISPR–Cas systems influence the prevalence of AMR genes, with CRISPR-containing isolates exhibiting a higher number of AMR genes for pathogens, such as *A. baumannii, E. faecium, P. aeruginosa,* and *S. aureus* [22, 28, 51, 52, 60]. High-resolution resistance profiles through shotgun sequencing have provided insights into the association between CRISPR–Cas systems and genome size variation, as well as their impact on AMR and defense systems [31]. Specific CRISPR–Cas applications have demonstrated impressive efficiencies in gene re-sensitization, such as gRNA_195 restoring fosfomycin sensitivity in bacteria, and pKJK5::csg[aacC1] significantly reduced the transformation efficiency of the targeted plasmid pHERD30T in *E. coli* [26, 29, 47]. The identification of resistance genes has uncovered 23 antibiotic resistance genes in all *A. baumannii* strains, highlighting a serious public health threat [28]. Optimization efforts have markedly enhanced editing efficiencies, with the CRISPR/Cas9 system effectively re-sensitizing multidrug-resistant (MDR) cells to antibiotics [24, 37, 38, 48, 55].

### Synthesis results

A qualitative synthesis approach was used because of the heterogeneity of the study designs and outcomes. Subgroup analyses were performed based on the different CRISPR/Cas systems and delivery methods. No meta-analysis was conducted because of the variability in study outcomes. The direction of the effects and heterogeneity among the studies are detailed in the results section, with specific attention given to discrepancies and variations in outcomes.

## Discussion

The findings presented in the table highlight the transformative potential of CRISPR/Cas technology in addressing AMR, particularly in the context of the WHO’s 2024 priority bacterial pathogens. The diversity of CRISPR–Cas systems employed, including CRISPR–Cas9, CRISPR–Cas12a, and CRISPR–Cas3, highlights the adaptability of this technology in addressing different bacterial targets and resistance mechanisms. CRISPR/Cas9’s precise double-strand break induction enables targeted gene editing, which is essential for inactivating resistance genes and restoring bacterial antibiotic susceptibility through genome modification [69]. CRISPR/Cas12 collateral cleavage activity and CRISPR/Cas13 RNA-targeting abilities further enhance our toolkit, offering innovative approaches to neutralize resistant bacterial strains contributing to AMR [8, 15]. This is supported by a previous study that emphasized the versatility of CRISPR–Cas system technology in targeting various resistance mechanisms and bacterial species [70]. The successful application of these systems across various delivery mechanisms, including bacteriophages, nanoparticles, and conjugative plasmids, further emphasizes the potential for tailored interventions depending on the specific pathogen and clinical setting [71]. This finding is consistent with studies that have extensively reported the challenges and opportunities of CRISPR–Cas delivery systems for antimicrobial applications [2, 72], highlighting the importance of optimizing delivery methods for in vivo applications, a challenge identified in our review [73].

These results align with previous findings that underscore the potential of CRISPR/Cas technology to revolutionize AMR management. The precision of CRISPR/Cas systems is critical for effectively targeting and editing genes, including resistance genes, without inducing off-target effects. These advancements in RNA design, system optimization, and specificity-enhancing modifications reduce unintended genetic alterations [2] and enable effective multiplexing and simultaneous gene editing, outperforming other methods, such as zinc finger nucleases (ZFNs) and transcription activator-like effector nucleases (TALENs), which often cause off-target effects [15]. By precisely targeting resistance determinants in priority pathogens, CRISPR/Cas technology can mitigate the spread of resistance and restore the effectiveness of existing antibiotics, a crucial step towards reinvigorating AMR resilience on a global scale [74]. This targeted approach offers a significant advantage over traditional broad-spectrum antibiotics, potentially mitigating the risk of further resistance development and preserving the efficacy of existing antimicrobial agents. These results align with those of another study highlighting the potential of CRISPR–Cas systems as promising alternatives to conventional antibiotics [75].

One of the most significant advantages of CRISPR–Cas technology over conventional antimicrobial strategies is its ability to selectively target resistant bacteria while sparing beneficial microbiota, thereby reducing dysbiosis [6]. Unlike broad-spectrum antibiotics, which disrupt the entire microbial community and predispose patients to secondary infections, CRISPR-based antimicrobials can eliminate pathogens without disturbing the commensal bacteria [76]. This feature was highlighted in the success of conjugative CRISPR–Cas9 antimicrobials by Sheng et al. [54], which effectively reduced antibiotic-resistant enteric pathogens without negatively impacting normal gut microbiota [54]. This precision-based approach may play a crucial role in restoring microbial balance, preventing opportunistic infections, and maintaining overall gut health, particularly in immunocompromised patients with leukemia. The ability of CRISPR–Cas to mitigate dysbiosis-associated complications, such as *Clostridioides difficile* infection, underscores its therapeutic superiority over traditional antibiotic therapies [77, 78]. Furthermore, CRISPR–Cas tools may serve as a promising strategy for engineering beneficial bacterial strains with enhanced colonization resistance against pathogenic species, strengthening host-microbiota interactions, and reducing infection risks [79].

CRISPR/Cas technologies can be employed in the detection and diagnosis of the WHO’s 2024 priority bacterial pathogens, presenting a promising strategy for managing high-risk multidrug-resistant (MDR) bacteria. Targeting resistance genes in pathogens, such as *Mycobacterium tuberculosis*, Enterobacterales, *Staphylococcus aureus,* and *E. coli*, with CRISPR/Cas can lead to enhanced susceptibility to first-line antibiotics and reduce the prevalence of MDR strains. In addition, CRISPR/Cas-based interventions can enhance the development of novel antimicrobial agents, addressing critical gaps in the current arsenal [73, 80]. While the efficacy of CRISPR/Cas systems in preclinical studies is promising, translating these findings into clinical practice involves addressing several challenges, such as the effective delivery of CRISPR/Cas components to target pathogens, minimizing off-target effects, and managing potential resistance to CRISPR/Cas interventions [81]. Most researchers employ plasmid electroporation to introduce specific systems into experimental bacterial cells; however, in vivo experimentation currently seems daunting. The phage delivery system is a sustainable approach with robust advantages over plasmid electroporation [80]. However, some studies have highlighted ongoing efforts to optimize delivery methods and enhance system specificity to optimize the potential of CRISPR/Cas technologies. The high efficiency of modalities, such as the near-complete elimination of targeted strains and their significant reduction in resistant bacterial populations, is promising [82].

The development of CRISPR-based diagnostic tools, as demonstrated in this review, represents a significant advancement in the treatment of AMR. The high sensitivity and specificity of these assays could revolutionize AMR surveillance and enable rapid, targeted interventions. This aligns with the findings of a previous study that explored the potential of CRISPR-based diagnostics for infectious diseases, emphasizing their potential for point-of-care testing (POCT) and rapid pathogen identification [83]. The inverse association observed between the presence of native CRISPR–Cas systems and the prevalence of AMR genes in some bacterial species [33] suggests that the CRISPR–Cas system is pivotal in limiting the horizontal gene transfer of AMR determinants. These findings have significant implications for antimicrobial stewardship and for public health. To improve the identification and treatment of resistant infections, cutting-edge CRISPR-based diagnostic and therapeutic methods must be incorporated into clinical practice. Health policy development should strengthen the use of systems to improve the early identification and targeted treatment of AMR pathogens [84].

### Implications of CRISPR/Cas systems for practice, policy, and future research in addressing WHO 2024 BPPL

The application of CRISPR/Cas systems to combat priority pathogens underscores the necessity of updated policy frameworks. The effective integration of these advanced technologies into national and global health policies can facilitate the development of targeted therapeutic strategies and surveillance systems for the disease. Policymakers should consider endorsing guidelines for the clinical use of CRISPR-based tools and establishing frameworks for their ethical deployment, including ensuring access to these technologies in resource-limited settings, where AMR is often the most prevalent [80, 85]. The deployment of CRISPR/Cas systems holds substantial promise for improving public health outcomes by offering precise and effective interventions against resistant bacteria. Studies have demonstrated enhanced detection and treatment of pathogens, such as NDM-producing bacteria and MRSA, leading to more effective control measures and reduced spread of infections in communities. Public health strategies should incorporate CRISPR technologies to bolster infection control and AMR mitigation efforts [2, 8]. CRISPR/Cas systems are versatile tools for addressing the rise in AMR on a global scale. For example, CRISPR/Cas9 can reverse antibiotic resistance in *E. coli* and target plasmids in multidrug-resistant strains of bacteria. The global health community must prioritize international collaboration to share CRISPR-based innovations and best practices [74, 86]. CRISPR/Cas systems are revolutionizing the management of resistant infections, improving treatment outcomes, and overcoming the limitations of current antimicrobial therapies. Clinical guidelines should incorporate CRISPR-based approaches for managing resistant infections, along with traditional therapies, to enhance patient care [87, 88]. The use of CRISPR/Cas technologies raises important ethical considerations, including the potential for off-target effects and the implications of genetic modifications. Ethical frameworks must be developed to guide the responsible use of these tools and ensure their safe and equitable application [14, 17]. Our review process, although comprehensive, may be limited by the inclusion criteria and publication biases inherent in the selected studies. Variations in study methodologies and reporting standards may affect the consistency of the results. In addition, reliance on preclinical studies may not fully capture the complexities and challenges of clinical applications.

### Strengths, limitations, and future directions

This comprehensive systematic review of CRISPR–Cas applications in AMR followed the PRISMA guidelines and utilized an extensive literature search across multiple databases. This aligns with the 2024 WHO Bacterial Priority Pathogens List, highlighting the versatility of CRISPR–Cas9, CRISPR–Cas12a, and CRISPR–Cas3 in targeting AMR genes and their role in virulence gene suppression. This review covers various resistance genes, evaluates various delivery mechanisms, and emphasizes microbiota preservation. This study evaluated both therapeutic and diagnostic applications, highlighting the potential of CRISPR–Cas in AMR surveillance, treatment, and antimicrobial stewardship. The inclusion of the Joanna Briggs Institute (JBI) tool ensured a rigorous evaluation of the included studies, improving the reliability of the findings. This study provides strategic recommendations for integrating CRISPR-based AMR interventions into clinical practice and public health policy, reinforcing their translational significance. These strengths make this review a valuable contribution to the ongoing fight against AMR.

Our review process, although comprehensive, may be limited by the inclusion criteria and publication biases inherent in the selected studies. Variations in study methodologies and reporting standards may affect the consistency of the results. In addition, reliance on preclinical studies may not fully capture the complexities and challenges of clinical applications. Variations in system efficiency, challenges in consistent delivery across different bacterial species, and the potential for off-target effects are further limitations. These limitations are in line with optimizing the delivery of CRISPR–Cas systems to target bacteria in vivo [89]. Future research should enhance the efficiency and specificity of delivery methods, especially for in vivo applications. Although high specificity has been reported, off-target effects are a concern. A careful guide to RNA design and thorough testing are essential to minimize unintended genomic modifications [90]. Scopus and PubMed were selected as the primary databases because of their extensive coverage of high-impact peer-reviewed biomedical and life sciences literature, particularly in the fields of microbiology, molecular biology, and gene editing. However, we acknowledge that excluding other databases, such as Web of Science (WoS) and Google Scholar, may introduce some limitations. Future research should consider integrating these databases to enhance the comprehensiveness of literature retrieval and mitigate potential publication bias.

**Fig. 3:**
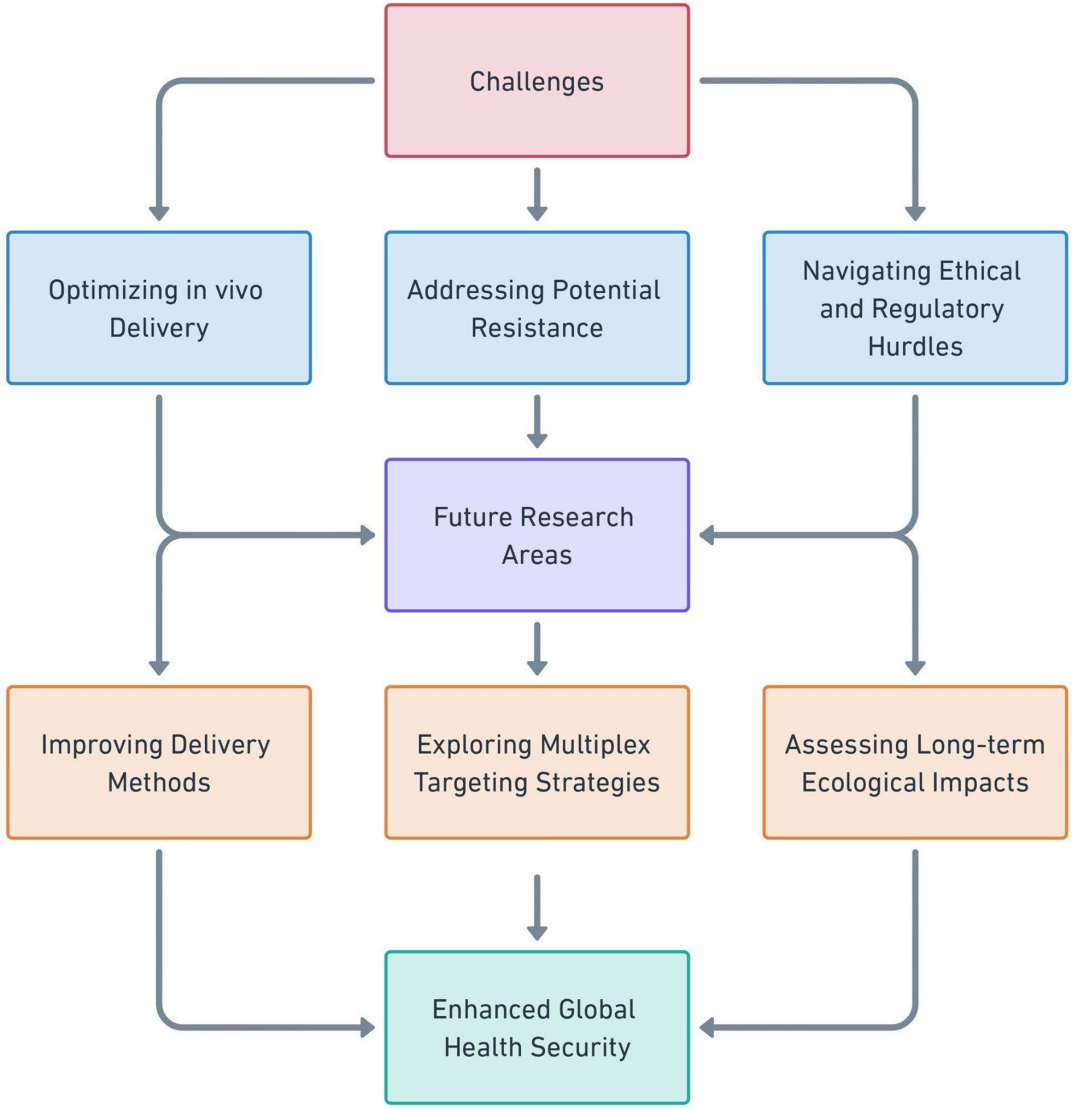
Recommendations for CRISPR–Cas technology

Strategic integration into existing AMR surveillance and response frameworks is essential to maximize the impact of CRISPR/Cas technologies on global health security. Collaboration among researchers, public health authorities, and policymakers will facilitate the development and deployment of CRISPR-based solutions tailored to high-priority pathogens. Moreover, investing in research to address the limitations of current systems while exploring innovative variants will further strengthen the understanding of the system and its potential to curb AMR [85]. Ethical considerations and regulatory challenges must be addressed to ensure the effective clinical implementation of this approach. Optimizing delivery systems, such as novel nanoparticle formulations and engineered bacteriophages, can enable bacterial population-specific targeting. Investigating synergies between CRISPR–Cas systems and other antimicrobial technologies, such as antimicrobial peptides and novel antibiotics, may lead to more effective therapies [75]. Long-term studies on the ecological impact and evolutionary consequences of CRISPR–Cas interventions in microbial communities are essential for understanding their broader implications (Fig. 3).

## Conclusion

This comprehensive review explores the potential of CRISPR–Cas technology to tackle AMR and contributes to the ongoing fight against the 2024 WHO-identified priority pathogens. Various CRISPR–Cas systems, such as CRISPR–Cas9, CRISPR–Cas12a, and CRISPR–Cas3, have shown versatility in targeting resistance genes and bacterial species. The reviewed studies highlighted systems capable of eradicating resistant strains, achieving near-complete elimination, and plasmid curing efficiencies of up to 100% in both in vitro and in vivo models. CRISPR-based diagnostics promise rapid and sensitive AMR detection with significant detection limits over conventional PCR methods. These advancements could revolutionize AMR surveillance and enable targeted interventions if integration bottlenecks are circumvented. CRISPR–Cas technology offers a promising approach for combating AMR, preserving antibiotic efficacy, and improving global health security. Utilizing these systems for the WHO 2024 BPPL will involve selectively targeting resistant bacteria or resensitizing them with first-line antibiotics. As research progresses, CRISPR–Cas is poised to play a pivotal role in addressing the global challenges posed by AMR.

## Supplementary Information

Supplementary material 1.

## Data Availability

No datasets were generated or analysed during the current study.
